# Supplementary material for: Griddient: a microfluidic array to generate reconfigurable gradients on-demand for spatial biology applications
Source: Commun Biol. 2023 Sep 9;6:925. doi: 10.1038/s42003-023-05282-3 (PMC10492845; doi:10.1038/s42003-023-05282-3)
Supplement: Supplementary file 2 — Supplementary Information [file 42003_2023_5282_MOESM2_ESM.pdf]

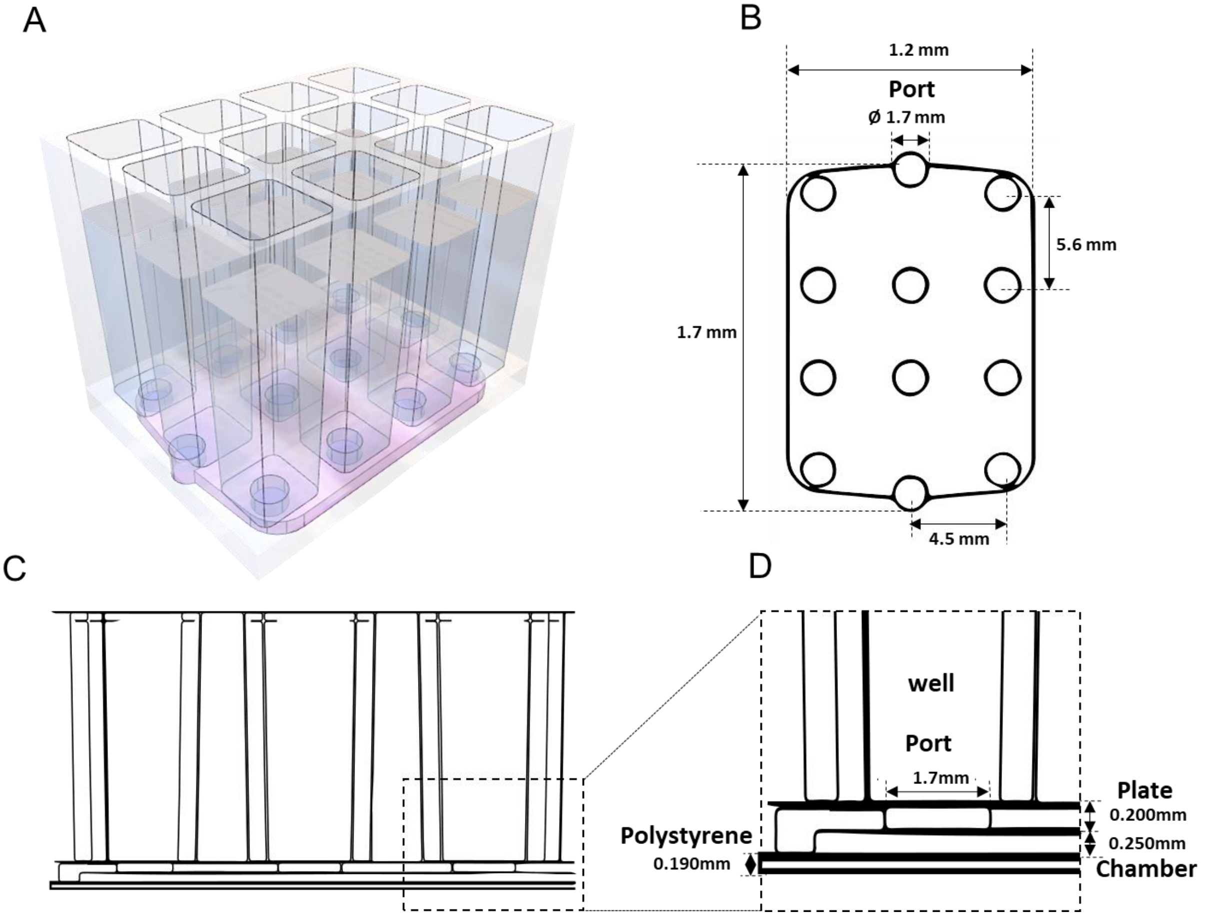

Supplementary Figure 1. Schematic representation of Griddient. A. Schematich 3D view of a Griddient chamber. B. Top view of a Griddient chamber. C Side view of a Griddient camber D. Magnification of the bottom part of a Griddient well.

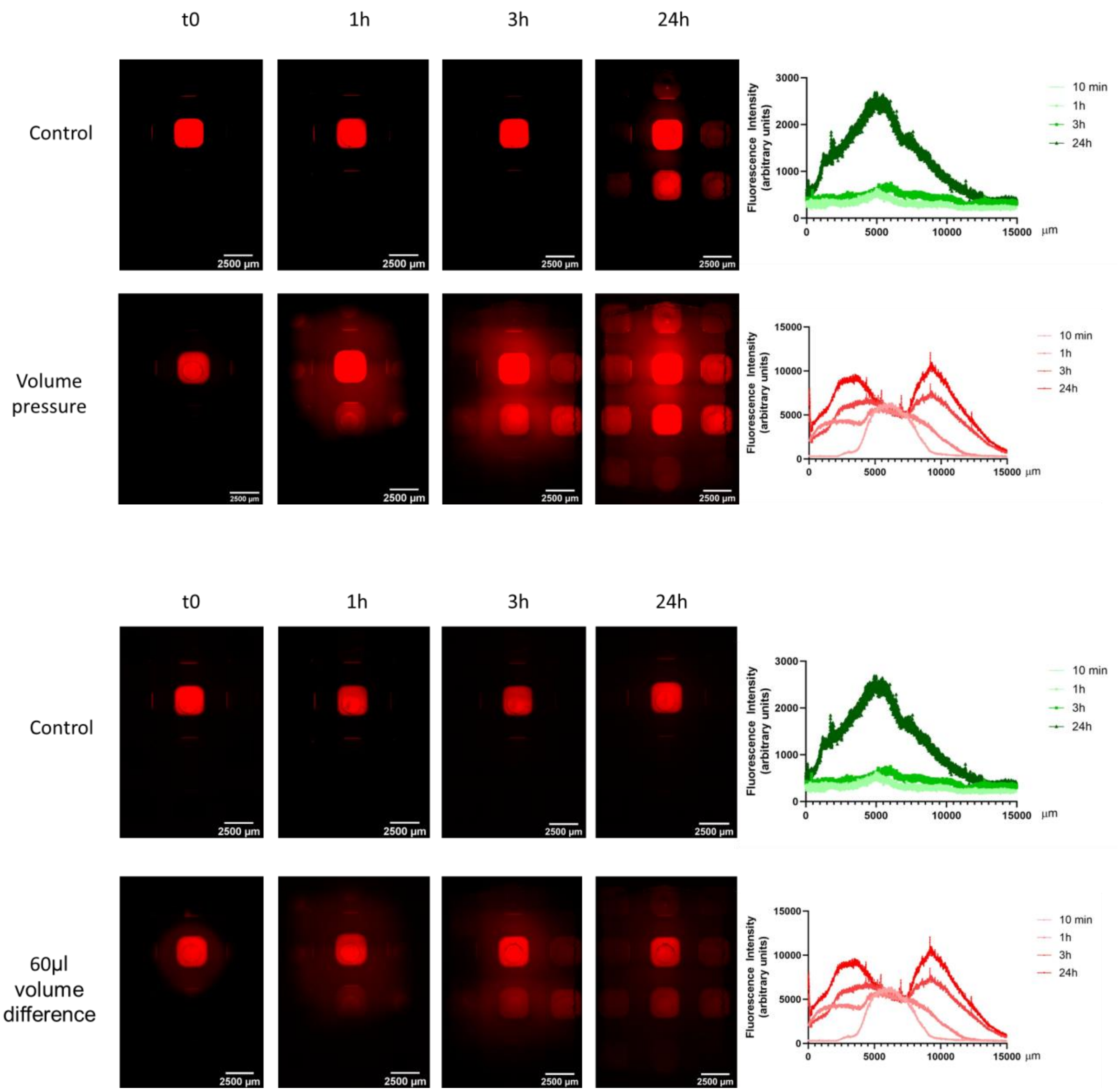

Supplementary Figure 2. Incidental pressure gradients. A. Representative images of 70 kDa TR-dextran diffusion in the Griddient in a pressure gradient situation. Griddient device was filled with collagen. After polymerization, 70 µl of 70 kDa TR-dextran were added in one of the central wells. The remaining wells were filled with 70 µl or 10µl of PBS for the control or the incidental pressure situation. We show representative images of the griddient at different time points as well quantification of dextran diffusion between the chamber for both situations. Results are plot as mean, n=3.
