# Supplementary material for: Griddient: a microfluidic array to generate reconfigurable gradients on-demand for spatial biology applications
Source: Commun Biol. 2023 Sep 9;6:925. doi: 10.1038/s42003-023-05282-3 (PMC10492845; doi:10.1038/s42003-023-05282-3)
Supplement: Supplementary file 3 — Reporting Summary [file 42003_2023_5282_MOESM3_ESM.pdf]

nature portfolio

Corresponding author(s): Jose Maria Ayuso

Last updated by author(s): Aug 17, 2023

Reporting Summary

Nature Portfolio wishes to improve the reproducibility of the work that we publish. This form provides structure for consistency and transparency in reporting. For further information on Nature Portfolio policies, see our Editorial Policies and the Editorial Policy Checklist.

Statistics

For all statistical analyses, confirm that the following items are present in the figure legend, table legend, main text, or Methods section.

n/a	Confirmed	
☐	☒	The exact sample size (*n*) for each experimental group/condition, given as a discrete number and unit of measurement
☐	☒	A statement on whether measurements were taken from distinct samples or whether the same sample was measured repeatedly
☐	☒	The statistical test(s) used AND whether they are one- or two-sided<br>*Only common tests should be described solely by name; describe more complex techniques in the Methods section.*
☐	☒	A description of all covariates tested
☐	☒	A description of any assumptions or corrections, such as tests of normality and adjustment for multiple comparisons
☐	☒	A full description of the statistical parameters including central tendency (e.g. means) or other basic estimates (e.g. regression coefficient) AND variation (e.g. standard deviation) or associated estimates of uncertainty (e.g. confidence intervals)
☐	☒	For null hypothesis testing, the test statistic (e.g. *F*, *t*, *r*) with confidence intervals, effect sizes, degrees of freedom and *P* value noted<br>*Give P values as exact values whenever suitable.*
☒	☐	For Bayesian analysis, information on the choice of priors and Markov chain Monte Carlo settings
☒	☐	For hierarchical and complex designs, identification of the appropriate level for tests and full reporting of outcomes
☒	☐	Estimates of effect sizes (e.g. Cohen's *d*, Pearson's *r*), indicating how they were calculated

Our web collection on statistics for biologists contains articles on many of the points above.

Software and code

Policy information about availability of computer code

Data collection	N/A
Data analysis	N/A

For manuscripts utilizing custom algorithms or software that are central to the research but not yet described in published literature, software must be made available to editors and reviewers. We strongly encourage code deposition in a community repository (e.g. GitHub). See the Nature Portfolio guidelines for submitting code & software for further information.

Data

Policy information about availability of data

All manuscripts must include a data availability statement. This statement should provide the following information, where applicable:

- Accession codes, unique identifiers, or web links for publicly available datasets
- A description of any restrictions on data availability
- For clinical datasets or third party data, please ensure that the statement adheres to our policy

The experimental data and the simulation results that support the findings of this study are available in Figshare under the project https://figshare.com/projects/Griddient_a_microfluidic_array_to_generate_reconfigurable_gradients_on-demand_for_spatial_biology_applications/175710. The identifiers for every experiment are the following 10.6084/m9.figshare.23961015
10.6084/m9.figshare.23961033

10.6084/m9.figshare.23961039
10.6084/m9.figshare.23961045
10.6084/m9.figshare.23961057

Research involving human participants, their data, or biological material

Policy information about studies with human participants or human data. See also policy information about sex, gender (identity/presentation), and sexual orientation and race, ethnicity and racism.

Reporting on sex and gender	n/a
Reporting on race, ethnicity, or other socially relevant groupings	n/a
Population characteristics	n/a
Recruitment	n/a
Ethics oversight	n/a

Note that full information on the approval of the study protocol must also be provided in the manuscript.

Field-specific reporting

Please select the one below that is the best fit for your research. If you are not sure, read the appropriate sections before making your selection.

☒ Life sciences ☐ Behavioural & social sciences ☐ Ecological, evolutionary & environmental sciences

For a reference copy of the document with all sections, see nature.com/documents/nr-reporting-summary-flat.pdf

Life sciences study design

All studies must disclose on these points even when the disclosure is negative.

Sample size	At least a sample size of three independent experiments was used to ensure the reproducibility of the data
Data exclusions	We did not exclude any data of the analysis. In case of data exclusion we will use the prism algorithm to identify outliers
Replication	Every experiment was conducted three times independently to ensure reproducibility. In the case of gene expression, several genes from the same of similar pathways were analyzed.
Randomization	positions of different conditions in the griddient were selected randomly and shuffled between independent experiments
Blinding	Conditions were named randomly with letters. data was analyzed and them matched with its condition to ensure blindness.

Reporting for specific materials, systems and methods

We require information from authors about some types of materials, experimental systems and methods used in many studies. Here, indicate whether each material, system or method listed is relevant to your study. If you are not sure if a list item applies to your research, read the appropriate section before selecting a response.

Materials & experimental systems

n/a	Involved in the study	
☒	☐	Antibodies
☐	☒	Eukaryotic cell lines
☒	☐	Palaeontology and archaeology
☒	☐	Animals and other organisms
☒	☐	Clinical data
☒	☐	Dual use research of concern
☒	☐	Plants

Methods

n/a	Involved in the study	
☒	☐	ChIP-seq
☒	☐	Flow cytometry
☒	☐	MRI-based neuroimaging

Eukaryotic cell lines

Policy information about cell lines and Sex and Gender in Research

Cell line source(s)	We used HCT-166 (adult male colon cancer), HUVEC (umbilical vein endothelial cells), Mesenchymal Stem cells (male). All cell lines were obtained directly from ATCC
Authentication	All cell lines were obtained directly from ATCC. This entity authentify all the cells lines in their dataset
Mycoplasma contamination	Mycoplasma is tested regularly by DAPI and qPCR.
Commonly misidentified lines (See ICLAC register)	n/a
